# Book Reviews

**Published:** 1894-10

**Authors:** 


### Book Reviews.

SMALL HOSPITALS. Establishment and Maintenance. By A. WORCESTER, A. M., M. D., and Suggestions for Hospital Architecture, with plans for a small hospital, by William Atkinson, Architect. Duodecimo, pp. vi.—114. Price, cloth, $1.25. New York: John Wiley & Sons, 53 East Tenth street. 1894.

In small cities it is often difficult to organize hospitals on a proper basis. Two serious obstacles always confront philanthropic men and women at the beginning of their new hospital movements. The first is, to reconcile the so-called different schools of medicine that must all be represented on the hospital staff; and second, the financial problem is always of the most serious import. When these two can properly be adjusted, the hospital is about as good as established. Valuable hints will be found in this book on these as well as on all other important questions connected with the subject.

It is written in a very agreeable manner and furnishes entertaining reading to all interested in the establishment of small hospitals.

The adjustment of the difficulties between the so-called different schools of medicine is treated of in a manner that will interest both profession and laity, as follows: "The chief difficulty concerns the so-called different schools. In common parlance and in common belief, there are at present in this country two schools of medical practice, one the old school or allopathic, and the other the homeopathic or new school. There are also other schools—the eclectic or Thompsonian, for instance—and there have more lately arisen, schools of faith-cure, mind-cure and of Christian Science healing. But hospitals are not likely to be vexed by any question concerning these smaller schools. The real difficulty comes in what may be called the homeopathic question.

"The first surprise in store for investigators into this question is the wrathy repudiation by all physicians of the term 'allopath.' The homeopathists cherish a theory of cure which is expressed by the word 'homeopathy.' So they, naturally enough, are willing to be called by that name. But physicians who do not happen to believe in any theory of cure naturally object to the name 'allopath,' which implies a belief, or a theory of practice, never countenanced, if, indeed, it ever existed. The early homeopathists called all others allopathists. That was, and is, considered a reproach by all who know what the word means. And that is why the designation is so indignantly repudiated by the ordinary physician.

"When it comes to the terms 'old school' and 'new school,' there again is found an unwillingness on the part of every physician to be called of the old school. The whole science of medicine, while as old as Galen, is still so rapidly improving that no member of the profession likes to be thought possessed of only time-honored methods.

"Thus, as the question is studied, the difficulty increases of finding acceptable terms in which to describe the very evident differences that divide the medical profession. Well may the managers ask, in despair, of the men who will not be called allopaths or old school doctors, 'What will you allow yourselves to be called?' Uufortunately, the answer will not be very satisfactory, the reason being that the great majority of physicians disclaim any particular system of practice. They feel themselves free to use any remedy or to adopt any methods or measures that promise relief to their patients; and so they want to be free from any prefix to their simple title of doctor of medicine, given them by their universities. They want to be called simply physicians or surgeons, just as lawyers are called attorneys or counsellors at law. But this wish of most doctors, not to have any extra designation, it must be allowed, is very inconvenient. And the public is so wedded to the use of the names 'old school,' and 'allopath,' and 'regular,' and the names are now used in such entire ignorance of the doctors' antipathy to them, that it is only on such occasions as that of a hospital's advent that vehement protests are evoked. No wonder the new managers are astonished at the outburst.

"And still, in spite of the inconvenience in not having a handy name for the medical practitioners in the community who are not homeopathists, it is, perhaps, not too much to ask of the managers that they shall respect the wishes of the common doctors in this matter, and speak of them simply as physicians and surgeons. This much is sure: by accepting the inconvenience and gratifying them in this matter, much will have been accomplished towards a satisfactory solution of the main question.

"Strange as it may at first appear, it is, nevertheless, true that most of the bitterness attached to the question will be found among the laity and not among the doctors. Indeed, the different kinds of doctors generally pull fairly well together. They visit in the same families; they are always willing to help each other in emergencies; and often they are very good friends. In some localities they consult together. But this is not generally the case."

This admirable exposition of the "pathy" question deserves to be read by every intelligent man and woman.

Annual of the Universal Medical Sciences. A Yearly Report of the Progress of the General Sanitary Sciences throughout the World. Edited by Charles E. Sajous, M. D., and seventy associate editors, assisted by over two hundred corresponding editors, collaborators and correspondents. Illustrated with chromo-lithographs, engravings and maps. Five volumes. The F. A. Davis Company, Publishers, Philadelphia, New York, Chicago and London. Australian Agency: Melbourne, Victoria. 1894.

This standard annual, the most complete in all its details, has gradually obtained the favor of the profession until it has become about the best consulted reference book extant. When it is a little delayed in its issue, subscribers become impatient, because they are anxious to find out what the fruits of the year in medical literature have been.

The series for 1894 is similar to its predecessors, being constructed on quite the same lines, both as to material and mechanical execution. To examine the literature of medicine that issues during the year, and to extract from the great mass that which is of value to preserve, is a Herculean labor, but Dr. Sajous and his associates have proved themselves more than equal to the task for six years before they undertook the present series. And to say that this one is fully up to the high standard already set, is only to render a just praise to a valuable work.

Some of the contributions are of a high order, though we refrain from making invidious distinction where all are so useful, not to say valuable.

It is a pleasure to note that the editor in his preface makes honorable mention of several members of his personal staff, who may be classed as editorial assistants. These are those who, though not contributing to the scientific part of the work, are yet indispensable coadjutors to its successful issue. They contribute to its mechanical excellence and to the promptness of its appearance from the press—two important factors in its success.

Every progressive physician has found the Annual so important an addition to his library that he does not need to be urged to subscribe for it. It is the rare exception to find it absent from a physician's library.

---

Diseases of the Skin; An Outline of the Principles and Practice of Dermatology. By Malcolm Morris, F. R. C. S., Surgeon to the Skin Department, St. Mary's Hospital, London, etc. In one 12mo volume of 572 pages, with nineteen chromo-lithographic figures and seventeen engravings. Cloth, $3.50. Philadelphia: Lea Brothers & Co. 1894.

The author of this book is recognized as a dermatologist of high repute all over the world, hence what he has to say will command respectful attention. His book is essentially a clinical treatise, and, therefore, appeals to the practical dermatologist. Moreover, it is cast in the most modern mold. It is well illustrated, with chromo-lithographic plates and wood-cuts distributed throughout the text.

An interesting part of the work is the chapter on artificial eruptions, which includes the various drug eruptions. The list of these is constantly being extended. Morris treats of the more important in detail, adding a table containing a summary of the eruptions caused by other drugs in common use. In this chapter vaccination eruptions are briefly considered.

This book is written for the general practitioner even more than for the specialist, and will be found a safe guide for him as well as an admirable manual for the student.

---

Asepsis in der Gynakologie und Geburtshuelfe. Von Dr. M. Saenger, ausserordentlicher Professor an der Universität, Leipzig, und Dr. W. Odenthal, Frauenarzt in Hanover, früher Assistenzarzt an Prof. Sänger's Heilanstalt. With two plates and forty-two illustrations in the text. Leipzig: C. G. Naumann. 1894.

This admirable little monograph is one of the numbers of the medical library for practising physicians similar to Davis's Physicians' Leisure Library, published in this country. It has two plates and forty-two illustrations in the text.

Sänger's opening sentence is admirable. He says: "Gynecology is the surgery of the sexual organs." This is substantially and practically true, and the same rigid rules of asepsis must apply to all gynecological procedures that belong to surgical operations. The same is equally true of all obstetrical work. When physicians learn that the lying-in room is to be made surgically clean and the patient prepared for her ordeal just as thoroughly as for the operating-table, then will there be less of puerperal sepsis and other conditions that produce ill-health or slow obstetric convalescence, and ophthalmia neonatorum will be an unknown disease.

We commend this little book to the careful study of obstetricians and gynecologists.

---

Transactions of the Indiana State Medical Society. Forty-fourth Annual Session, held in Indianapolis, Ind., May 11 and 12, 1893. Octavo, pp. 378. Indianapolis: Wm. B. Burford, Printer. 1893.

Among the State medical society transactions, that of Indiana must be accorded first rank. This statement holds good whether applied to the material published in the volume before us or to the mechanical execution of the book.

Nothing contributes more to the *esprit de corps* of the profession of medicine than a well-organized state medical society and the annual publication of a good volume of transactions. The practical subjects treated of in this volume, together with the able discussions had upon them, testify to the progressive spirit of our Indiana colleagues.

I. A Treatise on Diphtheria. By Dr. H. Bourges. Translated by E. P. Hurd, M. D. Physicians' Leisure Library. Price, cloth, 50 cents; paper, 25 cents. Detroit, Mich.: George S. Davis. 1894.

II. Treatment of Typhoid Fever. By D. D. Stuart, M. D., Lecturer on Clinical Medicine in the Jefferson Medical College of Philadelphia; Physician to the Medical Dispensary of the Episcopal Hospital, etc., etc. The Physicians' Leisure Library series, pp. 104. Price, paper, 25 cents; cloth, 50 cents. Detroit, Mich.: George S. Davis, 1893.

III. Where to send Patients Abroad for Mineral and other Water Cures and Climatic Treatment. By Dr. Thomas Linn, Doctor of Medicine, Faculty of Paris; Doctor of Medicine and Surgery, University of New York, etc., etc. Physicians' Leisure Library. Price, paper, 25 cents; cloth, 50 cents. Detroit, Mich.: George S. Davis. 1894.

These three books are among the latest issues of Davis's Physicians' Leisure Library series.

The treatise on diphtheria gives some of the more recent views on the disease both as to causation and treatment. The brief chapter on false diphtherias is especially interesting.

The treatment of typhoid fever is everywhere attracting attention, especially since the cold bath management is gaining ground. Dr. Stuart's little treatise, therefore, will command many readers.

Every physician has frequently occasion to send patients abroad either for change of climate or with reference to the use of mineral waters at the Spas. In Dr. Linn's manual will be found much information on this important subject.

---

Transactions of the Fifteenth Annual Meeting of the American Laryngological Association, held in the City of New York, May 22, 23 and 24, 1893. Octavo, pp. vi.—165. New York: D. Appleton & Company. 1894.

The transactions of this association, always full of interest to laryngologists, are especially so for the year 1893. The papers have already appeared in the *New York Medical Journal*, and so have had a large publication to the medical profession. Everyone who attempts to practise laryngology, whether a member of the association or not, will find it to his interest to subscribe for the annual transactions.

---

Index—Catalogue of the Library of the Surgeon-General's Office, United States Army. Authors and Subjects. Vol. XV., Universidad—Vzoroff. Quarto, pp. 842. Washington: Government Printing Office. 1894.

This valuable catalogue, now rapidly approaching completion, has occupied the attention of its compiler, Dr. John S. Billings, Deputy Surgeon-General U. S. Army for the past fifteen years. The probabilities are that one more volume will complete the series.

Volume XV. is somewhat smaller than its predecessors and contains 6,152 author-titles, representing 3,312 volumes and 4,235 pamphlets. It also includes 8,596 subject-titles of separate books and pamphlets and 35,667 titles of articles in periodicals. The total in the fifteen volumes, 163,605 author-titles, representing 80,806 volumes and 139,891 pamphlets; also 160,245 subject-titles of separate books and pamphlets, and 497,832 titles of articles in periodicals together with 4,335 portraits.

---

THE NURSE'S DICTIONARY OF MEDICAL TERMS AND NURSING TREATMENT. Compiled for the use of nurses and containing descriptions of the principal medical and nursing terms and abbreviations, instruments, drugs, diseases, accidents, treatments, physiological names, operations, foods, appliances, etc., etc., encountered in the ward or sick-room. By HONNER MORTEN, Author of Sketches of Hospital Life, How to Become a Nurse, etc. 32mo, pp. 139. Second edition. Price, $1.00. Philadelphia: W. B. Saunders, 925 Walnut street. 1894.

The importance of a handy glossary for nurses is so great that it is almost surprising that one has not been issued before. This edition corrects many deficiencies in the former one and considerably enlarges the little volume. It is still kept, however, within the proper limits of the pocket or handbag, so that it may always be in readiness. In the next edition it would be well to add the subject of pronunciation. Medical pronunciation is now becoming standardized, hence all dictionaries should pay attention to this important branch of medical learning. Every nurse should become possessed of this little book.

---

THE GRAPHIC HISTORY OF THE FAIR. Containing a sketch of International Expositions, a review of the events leading to the discovery of America and a history of the World's Columbian Exposition held in the City of Chicago, State of Illinois, May 1 to October 31, 1893. Describing the notable features in the several departments, the sculpture of buildings and grounds, the department edifices, the state and foreign buildings and pavilions, the exhibits, etc., etc. With nearly 1,000 illustrations. Chicago: The Graphic Company, 358 Dearborn street. 1894.

Many books have been published commemorating the Columbian Exposition, some of which are constructed on the heroic order, and most of which are incomplete both in illustration and text. The one before us, known as the Graphic History of the Fair, possesses many points of superiority, among which may be mentioned conciseness, completeness of detail, profuseness of illustration, convenience in size and cheapness.

Almost everyone desires to possess some memento of the great World's Fair held in Chicago in 1893, and we have seen no work that affords us more satisfaction than this one. The illustrations are especially to be commended for their artistic perfection, while the text is well written, in plain type, and the paper of fine finish and good weight.

PART I., ESSENTIALS OF REFRACTION AND DISEASES OF THE EYE. By EDWARD JACKSON, A. M., M. D., Professor of Diseases of the Eye in the Philadelphia Polyclinic and College for Graduates in Medicine, etc., etc. Part II., Essentials of Diseases of the Nose and Throat. By E. BALDWIN GLEASON, M. D., Surgeon in charge of the Nose, Throat and Ear Department of the Northern Dispensary; Assistant in the Ear Department of the Philadelphia Polyclinic and College for Graduates in Medicine, etc., etc. Two volumes in one, crown 8vo, 290 pages, profusely illustrated. Price, cloth, $1; interleaved for notes, $1.25. Philadelphia: W. B. Saunders, 925 Walnut street. 1894.

Many of the intricate questions relating to refraction are here treated of in a clear manner; so, too, with those diseases of the eye of the commoner varieties. Laryngology occupies the second half of the book and is well handled. It is truly a treatise on essentials and is one of the best of Saunders' Question Compends.

---

A CLINICAL MANUAL. A Guide to the Practical Examination of the Excretions, Secretions and the Blood, for the Use of Physicians and Students. By ANDREW MACFARLANE, A. B., M. D, Instructor in Neurology and Diseases of the Chest in the Albany Medical College; Physician to St. Peter's Hospital Out-patient Department, and Physician to Albany's Hospital for Incurables. Duodecimo, pp. xii.—139. Price, $1.25. New York: G. H. Putnam's Sons, 27 West Twenty-third street. 1894.

The author of this little book is to be congratulated upon the manner in which he has grouped so much information into such a narrow limit of book space. No physician can practise medicine successfully unless he employs all intelligent methods for the interpretation of the phenomena of disease. He must interrogate the excretions and secretions in the closest manner, for they often aid in diagnosticating obscure maladies.

Accurate diagnosis forms the foundation of successful treatment. The diagnostician must be at once a microscopist, chemist and bacteriologist. We do not mean, of course, that he must be an expert in all these several branches of laboratory work, but he must have some knowledge of such procedures as can be carried on in his office with a simple apparatus.

This manual by MacFarlane furnishes a valuable aid to the kind of work indicated.

---

### BOOKS RECEIVED.

A Practical Manual of Mental Medicine. By Dr. E. Régis, Professor of Mental Diseases, Faculty of Medicine, Bordeaux, formerly Chief of Clinique of Mental Diseases, Faculty of Medicine, Paris. With a preface by M. Benjamin Ball, Clinical Professor of Mental Diseases, Faculty of Medicine, Paris. Second edition, thoroughly revised and largely rewritten. Authorized translation by H. M. Bannister, A. M., M. D., late Senior Assistant Physician Illinois Eastern Hospital for the Insane. With introduction by the author. Pp. xvi.—692. Utica. N. Y.: Press of American Journal of Insanity. 1894.

Attfield's Chemistry. Fourteenth edition. Chemistry: General, Medical and Pharmaceutical; including the Chemistry of the U. S. Pharmacopeia. A Manual of the General Principles of the Science and their Application to Medicine and Pharmacy. By John Attfield, M. A., Ph. D., F. I. C., F. C. S., F. R. S., etc., Professor of Practical Chemistry to the Pharmaceutical Society of Great Britain, etc. Fourteenth edition, specially revised by the author for America to accord with the new U. S. Pharmacopeia. In one handsome royal 12mo volume of 794 pages, with 88 illustrations. Cloth, $2.75; leather, $3.25. Philadelphia: Lea Brothers & Co. 1894.

Transactions of the Medical and Chirurgical Faculty of the State of Maryland. Ninety-sixth annual session, held at Baltimore, Md., April, 1894, also semi-annual session, held at Annapolis, Md., November, 1893.

Transactions of the Texas State Medical Association. Twenty-sixth annual session, Austin, Texas, April 24, 25, 26 and 27, 1894.

Fifteenth Annual Report of the State Board of Health of Illinois, for the year ending December 31, 1892. With an appendix containing the report on medical education and medical colleges. Revised to December 31, 1893. Springfield, Illinois: W. H. Rokker, State Printer and Binder. 1894.

Hare's Text-Book of Practical Therapeutics. A Text-Book of Practical Therapeutics; with especial reference to the Application of Remedial Measures to Disease and their Employment upon a Rational Basis. By Hobart Amory Hare, M. D., Professor of Therapeutics and Materia Medica in the Jefferson Medical College of Philadelphia. With special chapters by Drs. G. E. de Schweinitz, Edward Martin and Barton C. Hirst. New (fourth) edition, thoroughly revised and much enlarged. In one octavo volume of 740 pages. Cloth, $3.75; leather, $4.75. Philadelphia: Lea Brothers & Co. 1894.

Flint's Practice of Medicine. A Treatise on the Principles and Practice of Medicine. Designed for the use of Students and Practitioners of Medicine. By Austin Flint, M. D., LL. D., Professor of the Principles and Practice of Medicine and of Clinical Medicine in Bellevue Hospital Medical College, N. Y. New (seventh) edition, thoroughly revised by Frederick P. Henry, M. D., Professor of the Principles and Practice of Medicine in the Woman's Medical College of Pennsylvania, Philadelphia. In one very handsome octavo volume of 1143 pages, with illustrations. Cloth, $5.00; leather, $6.00. Philadelphia: Lea Brothers & Co. 1894.

Young's Orthopedic Surgery. A Manual of Orthopedic Surgery for Students and Practitioners. By James K. Young, M. D., Instructor in Orthopedic Surgery, University of Pennsylvania, Philadelphia. In one octavo volume of 446 pages, with 285 illustrations. Cloth, $4.00; leather, $5.00. Philadelphia: Lea Brothers & Co. 1894.

Bread from Stones. A New and Rational System of Land Fertilization and Physical Regeneration. Translated from the German. Duodecimo, pp. 135. Price, 25 cents. Philadelphia, Pa.: A. J. Tafel, 1011 Arch street. 1894.

A System of Legal Medicine. By Allan McLane Hamilton, M. D., Consulting Physician to the Insane Asylums of New York City, etc., etc., and Lawrence Godkin, Esq., of the New York Bar. With twenty-seven collaborators. Illustrated. Volume I. Large 8vo, pp. 657. Price per volume, cloth, $5.50 ; full sheep, $6.50. New York: E. B. Treat, 5 Cooper Union. 1894.

Materia Medica, Pharmacology and Therapeutics. By W. Hale White, M. D., F. R C. P., Physician to, and Lecturer on, Materia Medica and Therapeutics at Guy's Hospital, London ; Examiner in Materia Medica to the Conjoint Board of England, etc. Edited by Reynold W. Wilcox, M. A., M. D., LL. D., Professor of Clinical Medicine and Therapeutics at the New York Post-Graduate Medical School and Hospital ; Visiting Physician to St. Mark's Hospital, etc. Second American edition, thoroughly revised. Small 8vo, pp. 661. Price, $3.00. Philadelphia : P. Blakiston, Son & Co., 1012 Walnut street. 1894.

The Medical Register of New York, New Jersey and Connecticut. For the year commencing June 1, 1894. Published under the supervision of the New York Medico-Historical Society. John Shrady, M. D., Editor. Volume XXXII. Price, $2.10. G. P. Putnam's Sons, New York, 27 West Twenty-third street ; London, 24 Bedford street, Strand. 1894.

Transactions of the American Gynecological Society. Volume XIX. For the year 1894. Small 8vo, pp. xliii.—363. Philadelphia : Wm. J. Dornan, Printer. 1894.

Transactions of the Medical Society of the State of New York. For the year 1894. Octavo, pp. 507. Published by the Society. 1894.

Abbott's Bacteriology. The Principles of Bacteriology : A Practical Manual for Students and Physicians. By A. C. Abbott, M. D., First Assistant, Laboratory of Hygiene, University of Pennsylvania, Philadelphia. New (second) edition. In one 12mo volume of 472 pages, with ninety-four illustrations, of which seventeen are colored. Cloth, $2.75. Philadelphia : Lea Brothers & Co. 1894.

Essentials of the Diseases of the Ear, arranged in the form of Questions and Answers. Prepared especially for Students of Medicine and Post-Graduate Students. By E. B. Gleason, S. B., M. D., Clinical Professor of Otology, Medico-Chirurgical College, Philadelphia ; Surgeon in Charge of the Nose, Throat and Ear Department of the Northern Dispensary, Philadelphia. Saunders' Question-Compends, No. 24. Price, $1.00. Philadelphia : W. B. Saunders, 925 Walnut street. 1894.

A Manual of Human Physiology, Prepared with Special Reference to Students of Medicine. By Joseph H. Raymond, A. M., M. D., Professor of Physiology and Hygiene in the Long Island College Hospital, and Director of Physiology in the Hoagland Laboratory. Saunders' New Aid Series. Small 8vo, pp. 382. With 102 illustrations in text and four full-page colored plates. Price, $1.25 net. Philadelphia : W. B. Saunders, 925 Walnut street. 1894.

---

Christian scientists must now procure certificates from the State Board of Health in Nebraska before they are allowed to practise their peculiar healing powers upon the inoffensive and credulous public. Not a bad law for California.—*Pacific Medical Journal.*

